# Impaired Mitophagy: A New Potential Mechanism of Human Chronic Atrial Fibrillation

**DOI:** 10.1155/2020/6757350

**Published:** 2020-10-01

**Authors:** Shuang Zhou, Weiran Dai, Guoqiang Zhong, Zhiyuan Jiang

**Affiliations:** Department of Cardiology, The First Affiliated Hospital of Guangxi Medical University, Guangxi Cardiovascular Institute, Nanning, Guangxi, China

## Abstract

Mitophagy is an autophagic response and plays essential roles in survival, development, and homeostasis of cells. It has been reported that mitophagic dysfunction is involved in several cardiovascular diseases. However, the effect of mitophagy on atrial fibrillation (AF) is still unknown. Therefore, we investigated the exact role of mitophagy in human chronic AF. Western blot was used to detect the protein abundance. The mitochondrial morphology and structure were observed by transmission electron microscopy. Immunofluorescent stainings were performed to analyze colocalization of mitochondria with autophagosomes or lysosomes. Totally, 43 patients with valvular heart disease undergoing cardiac surgery were selected, including 21 patients with chronic AF. Comparing with the sinus rhythm (SR) group, we found the size and number of mitochondria in atrial myocytes of patients with AF increased significantly. In addition, expression of LC3B II and LC3B II/LC3B I ratio was significantly decreased in the AF group. Moreover, the expression of p62 was markedly elevated in the AF group compared with that in the SR group. The results of immunofluorescence staining and western blot showed an enhanced expression of Cox IV in the AF group. Dual immunofluorescent stainings revealed that mitophagy defect in atrial myocytes of patients with AF resulted from dysfunction in the process of delivery of mitochondria into autophagosomes. For the first time, impaired mitophagy, during the phagocytosis of mitochondria, is associated with human chronic AF. Mitophagy could be a potential therapeutic target for AF.

## 1. Introduction

Atrial fibrillation (AF) is the most common arrhythmia, which increases the risk of stroke and heart failure and is associated with significantly increased mortality [1, 2]. However, the pathogenesis of AF is still not fully understood, and the intervention is challenging. Mitochondria are key players in cardiomyocyte energy metabolism, redox state regulation and apoptosis. Recently, increasing evidence reveals that mitochondrial dysfunction provides an arrhythmogenic substrate for initiation and perpetuation of AF, such as oxidative stress, energy metabolic dysregulation, and calcium homeostasis imbalance [3–5].

Autophagy is a conserved cellular pathway that is characterized by the engulfment of cargo into double-membrane autophagosomes, which then fuse with lysosomes for degradation. Autophagy plays essential roles in survival, development, and homeostasis of cells. To ensure survival, the cell uses autophagy to selectively digest protein aggregates, oxidized lipids, and damaged organelles [6, 7]. Mitophagy is an autophagic response that specifically targets damaged, and hence potentially cytotoxic, mitochondria, which plays a crucial role in the mitochondrial quality control and maintenance of mitochondrial function [8]. It has been reported that mitophagic dysfunction is involved in several cardiovascular diseases in the animal model, such as atherosclerosis, cardiac hypertrophy, and cardiomyopathy [8]. Recently, some studies have indicated that autophagy dysfunction contributes to AF [9–12]. However, the effect of mitophagy on AF is still unknown. Therefore, in this study, we investigated the exact role of mitophagy in human chronic AF.

## 2. Materials and Methods

### 2.1. Collection of Human Atrial Samples

A total of 43 patients with valvular heart disease undergoing cardiac surgery were selected and divided into a chronic AF group (21 cases with long-standing persistent AF) and a sinus rhythm (SR) group (22 cases; Table 1). The diagnosis of AF was achieved by evaluating medical records and 12-lead electrocardiogram findings. Those who had hypertension, diabetes mellitus, coronary heart disease, infective endocarditis, active rheumatism, pulmonary disease, hyperthyroidism, or autoimmune disease were excluded from this study. The protocol was in accordance with the Helsinki Declaration and was approved by the Human Ethics Committee of the First Affiliated Hospital of Guangxi Medical University (Guangxi, China). All patients enrolled in this study provided written informed consent.

Right atrial appendage (RAA) tissues weighing about 100 mg were removed at the beginning of the surgical interventions under extracorporeal circulation. A portion of the tissues was fixed in 2.5% glutaraldehyde for ultrastructure analysis under transmission electron microscopy. The remainder of the tissues were snap-frozen in liquid nitrogen for protein isolation and frozen sections to perform an immunofluorescence staining.

### 2.2. Western Blot

Total proteins were extracted from tissues by RIPA buffer (Beyotime Institute of Biotechnology, Shanghai, China) with PMSF (Sigma-Aldrich, St. Louis, Mo., USA). Protein concentration was determined with a BCA protein assay kit (Beyotime Institute of Biotechnology, Shanghai, China). The proteins were boiled with 5X SDS-PAGE sample loading buffer (Beyotime Institute of Biotechnology, Shanghai, China) and separated by 8%, 10%, or 12% SDS-PAGE, followed by transfer onto a 0.22 *μ*m polyvinylidene difluoride membrane (EMD Millipore, Billerica, MA, USA) using the Mini Trans-Blot electrophoretic transfer cell system (Bio-Rad Laboratories, Inc., Hercules, CA, USA). The membranes were blocked for 1 h at room temperature with 5% nonfat milk in TBST (20 mM Tris-HCl, 0.5 M NaCl, 0.1% Tween 20) and incubated with anti-COX II antibody (Abcam, Cambridge, UK) diluted at 1 : 5,000, anti-COX IV antibody (Cell Signaling Technology, Danvers, MA, USA) diluted at 1 : 1,000, anti-LC3B antibody (Abcam, Cambridge, UK) diluted at 1 : 2,000, or anti-p62 antibody (Cell Signaling Technology, Danvers, MA, USA) diluted at 1 : 1,000 overnight at 4°C. Subsequently, the membranes were incubated with IRDye 700CW goat anti-rabbit or anti-mouse IgG (LI-COR Biotechnology, Lincoln, NE, USA) diluted at 1 : 10,000 for 1 hour at room temperature. The signals were visualized and quantified with the Odyssey system (LI-COR Biosciences, St. Charles, USA). Protein band intensities were expressed relative to glyceraldehyde-3-phosphate dehydrogenase (GAPDH), which was incubated with anti-GAPDH antibody (Abcam, Cambridge, UK) diluted at 1 : 10,000 overnight at 4°C.

### 2.3. Immunofluorescence Staining

The frozen RAA tissues were embedded in the optimum cutting temperature (OCT) compound and sectioned at −25°C in a cryostat. Sections (5 *μ*m in thickness) were fixed with 4% polyformaldehyde for 15 minutes at room temperature. The sections were permeabilized with 0.1% TritonX-100 for 20 minutes followed by blocking with 10% goat serum for 1 hour at room temperature. Then, the sections were incubated with anti-Cox IV antibody (Cell Signaling Technology, Danvers, MA, USA) diluted at 1 : 200, anti-LAMP-1 antibody (Cell Signaling Technology, Danvers, MA, USA) diluted at 1 : 200, or anti-LC3B antibody (Abcam) diluted at 1 : 100 overnight at 4°C. Subsequently, the sections were incubated with Alexa Fluor 488-conjugated goat anti-mouse antibody (Cell Signaling Technology, Danvers, MA, USA) diluted at 1 : 500, or Alexa Fluor 594-conjugated goat anti-rabbit antibody (Cell Signaling Technology, Danvers, MA, USA) diluted at 1 : 500 for 1 hour at room temperature. DAPI (Solarbio Science & Technology, Beijing, China) was used to stain the cell nuclei. Finally, the fluorescence images were captured by using a laser scanning microscope and further analyzed by ImagePro Plus 6.0 (Media Cybernetics, Inc., Bethesda, MD, USA).

### 2.4. Mitochondrial Morphology Analysis

The mitochondrial morphology and structure were observed by transmission electron microscopy. The RAA tissues were cut into 1 × 1 × 1 mm sized pieces and fixed with 2.5% glutaraldehyde for 4 hours. Then, the samples were washed with 0.1 M phosphate-buffered saline (PBS) and postfixed by 1% osmium tetroxide solution for 2 hours. Subsequently, the samples were dehydrated in an ethanol gradient series (100, 90, 80, 75, and 50%) and 100% acetone, immersed in propylene oxide, and embedded in epoxy resin at 60°C. Ultrathin sections were obtained by using a Leica EM UC6rt ultramicrotome (Leica Microsystems, Inc., Wetzlar, Germany) and were doubly stained with 0.1% uranyl acetate and 3% lead citrate solution. The sections were observed and photographed by using a transmission electron microscope (H-7650, Hitachi, Ltd., Tokyo, Japan). A further statistical analysis was performed by Image J _v1.8.0 (Media Cybernetics, Inc., Bethesda, MD, USA).

### 2.5. Detection of Colocalization of Mitochondria with Autophagosomes or Lysosomes

Dual immunofluorescent stainings were performed to analyze colocalization of mitochondria with autophagosomes or lysosomes. As we know, Cox IV is an inner mitochondrial membrane protein. We used Cox IV to identify mitochondria. Cox IV was labeled with Alexa Fluor 488-conjugated goat anti-mouse antibody showing green fluorescence, which represented mitochondrial mass and localization. LAMP-1 that is an integral glycoprotein residing in lysosomes was used for identification of lysosomes [13]. LAMP-1 was labeled with Alexa Fluor 594-conjugated goat anti-rabbit antibody showing red fluorescence, which represented lysosomal mass and localization. LC3 was labeled with Alexa Fluor 594-conjugated goat anti-rabbit antibody showing red fluorescence and used for identification of autophagosomes. The merged image of Cox IV and LC3 that showed yellow fluorescence indicated the engulfment of mitochondria into autophagosomes. The merged image of Cox IV and LAMP-1 that showed yellow fluorescence indicated the infusion of autophagosomes enclosing mitochondria with lysosomes. The fluorescence images were captured by using a laser scanning microscope, and the fluorescence intensity was quantified by ImagePro Plus 6.0 (Media Cybernetics, Inc., Bethesda, MD, USA).

### 2.6. Statistical Analysis

The continuous data are presented as mean ± standard deviation (SD). The discrete data are shown as percentages. The continuous variables between 2 groups were analyzed by the unpaired Student's *t*-test. The discrete variables between 2 groups were analyzed by the Chi-square test. *p* < 0.05 was considered to indicate a statistically significant difference. All statistical analyses were performed using SPSS 16.0 (SPSS Inc., Chicago, IL, USA).

## 3. Results

### 3.1. Impaired Cardiac Autophagy in Patients with Chronic Atrial Fibrillation

**Figure 1:**
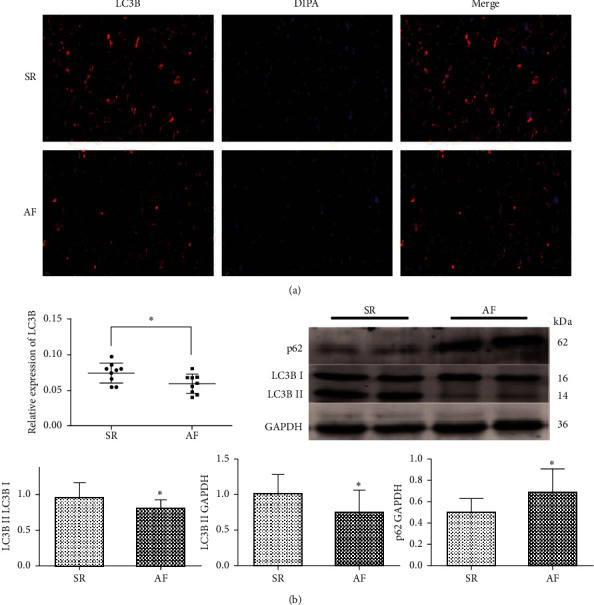
Impaired cardiac autophagy in patients with chronic AF. (a) Representative immunofluorescence images and semiquantitative analysis of LC3B in RAA (*n* = 9 per group). (b) Representative western blots and quantification of protein expressions of LC3B and p62 in RAA (the SR group, *n* = 16; the AF group, *n* = 18). Scale bar = 20 *μ*m. ^*∗*^*p* < 0.05 vs the SR group. SR, sinus rhythm; AF, atrial fibrillation; RAA, right atrial appendage.

To explore the role of autophagy on AF, the autophagy markers LC3B and p62 were measured in the SR group and the AF group, respectively. The results of immunofluorescence staining suggested a decreased expression of LC3B in the AF group (*p* = 0.046; Figure 1(a)). Consistently, the results of western blot revealed that expression of LC3BII and the LC3BII/LC3BI ratio were both significantly decreased in the AF group compared with that in the SR group (*p* = 0.013 and *p* = 0.03, respectively; Figure 1(b)). Moreover, the expression of p62 was markedly elevated in the AF group compared with that in the SR group (*p* = 0.014; Figure 1(b)). These results indicated that there was a cardiac autophagy defect in the patients with chronic AF.

### 3.2. Mitochondria Accumulation and Increased Autophagic Vacuoles in Atrial Myocytes of Patients with Chronic AF

**Figure 2:**
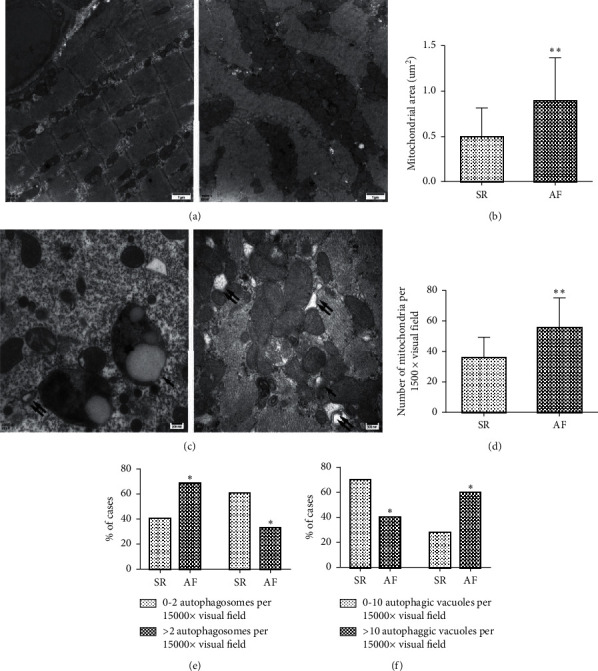
Mitochondria accumulation and increased autophagic vacuoles in atrial myocytes of patients with chronic AF. (a) Representative electron microscopic images of mitochondria in RAA (*n* = 5 per group, scale bar = 1 *μ*m). The asterisk indicates typical mitochondrial. (b, d) Semiquantitative analysis of mitochondrial area and quantity of mitochondria in RAA. (c) Representative electron microscopic images of autophagosomes and autophagic vacuoles in RAA (*n* = 5 per group, scale bar = 200 nm). The autophagosome containing mitochondria is marked with a single arrow. The autophagic vacuole is marked with double arrows. (e, f) Semiquantitative analysis of autophagosomes and autophagic vacuoles in RAA. ^*∗*^*p* < 0.05, ^*∗∗*^*p* < 0.01 vs the SR group. SR, sinus rhythm; AF, atrial fibrillation; RAA, right atrial appendage.

The differences of mitochondrial morphology and number between the SR group and the AF group were observed by transmission electron microscopy. We found that the mitochondria were larger, and their numbers were obviously increased in atrial myocytes of patients in the AF group, compared with those in the SR group (*p* = 0.001 and *p* = 0.001, respectively; Figure 2(a)). In addition, there was a significant increase in autophagic vacuoles and decrease in infusion autophagosomes with lysosomes (*p* = 0.023 and *p* = 0.047, respectively; Figure 2(b)). These results demonstrated that the mitophagy in atrial myocytes of patients with chronic AF may be impaired.

### 3.3. Defective Mitophagy in Atrial Myocytes of Patients with Chronic AF

**Figure 3:**
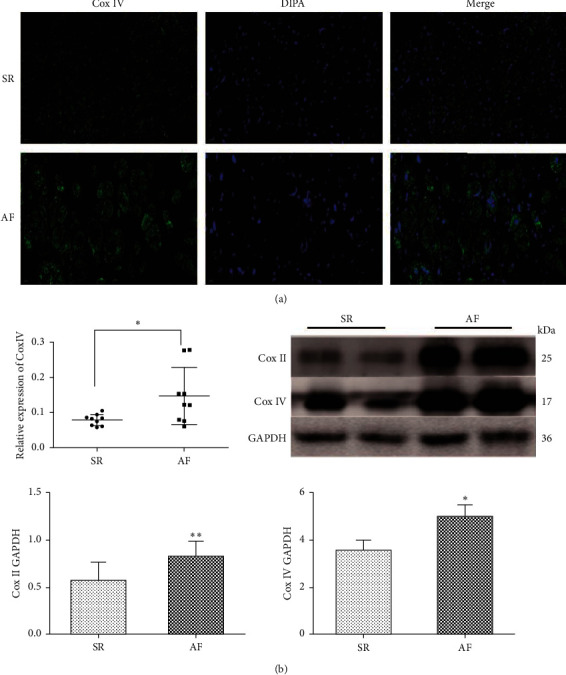
Defective mitophagy in atrial myocytes of patients with chronic AF. (a) Representative immunofluorescence images and semiquantitative analysis of Cox IV in RAA (*n* = 9 per group, scale bar = 20 *μ*m). (b) Representative western blots and quantification of protein expressions of Cox II and Cox IV in RAA (the SR group, *n* = 14; the AF group, *n* = 18). ^*∗*^*p* < 0.05, ^*∗∗*^*p* < 0.01 vs the SR group. SR, sinus rhythm; AF, atrial fibrillation; RAA, right atrial appendage.

Mitophagy is an autophagic response and plays a crucial role in the mitochondrial quality control process. To observe the change in mitophagy in patients with chronic AF, Cox II and Cox IV, which are localized to the inner mitochondrial membrane and are used as marks for mitophagy [14, 15], were determined in the SR group and the AF group, respectively. The results of immunofluorescence staining showed an enhanced expression of Cox IV in the AF group (*p* = 0.024; Figure 3(a)). Consistently, the results of western blot showed that the expression of Cox II and Cox IV was significantly increased in the AF group, compared with that in the SR group (*p* = 0.001 and *p* = 0.032, respectively; Figure 3(b)). These findings further verified that there was a defective mitophagy in atrial myocytes of patients with chronic AF.

### 3.4. Decreased Engulfment of Mitochondria into Autophagosomes in Atrial Myocytes of Patients with Chronic AF

**Figure 4:**
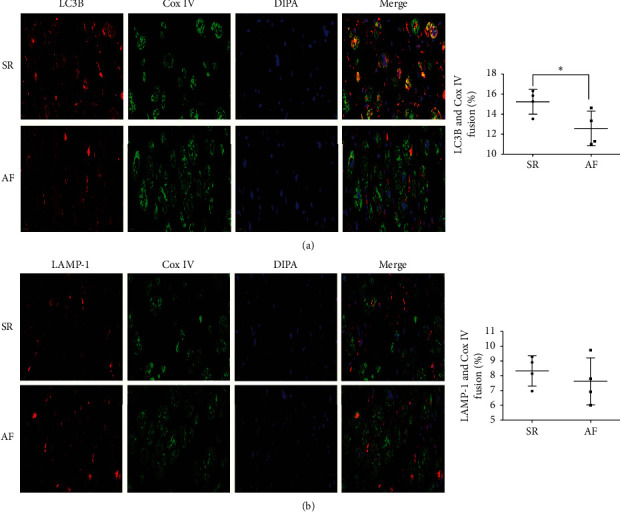
Decreased engulfment of mitochondria into autophagosomes in atrial myocytes of patients with chronic AF (*n* = 4 per group, scale bar = 20 *μ*m). (a) Representative dual immunofluorescence images and semiquantitative analysis colocalization of Cox IV and LC3B in RAA. (b) Representative dual immunofluorescence images and semiquantitative analysis colocalization of Cox IV and LAMP-1 in RAA. ^*∗*^*p* < 0.05 vs the SR group. SR, sinus rhythm; AF, atrial fibrillation; RAA, right atrial appendage.

To investigate the underlying mechanism of mitophagy defect in chronic AF patients, the mitophagy flux was detected by dual immunofluorescent stainings. The results showed that the yellow fluorescence intensity caused by merge of Cox IV and LC3 was obviously decreased in the AF group compared with that in the SR group (*p* = 0.046; Figure 4(a)). However, the yellow fluorescence intensity caused by merge of Cox IV and LAMP-1 had no significant difference between the SR group and the AF group (*p* = 0.485; Figure 4(b)). These findings suggested that mitophagy defects in atrial myocytes of patients with chronic AF may result from dysfunction in the process of delivery of mitochondria into autophagosomes.

## 4. Discussion

In the present study, we found impaired mitophagy in patients with chronic AF and further demonstrated that dysfunction in the process of engulfment of mitochondria into autophagic vesicles results in defective mitophagy. To the best of our knowledge, this is the first study to report that impaired mitophagy is associated with human chronic AF. Mitophagy could be a potential therapeutic target for AF.

In this study, a decreased LC3BII level and LC3BII/LC3BI ratio were observed in RAA tissues of patients with chronic AF. Meanwhile, an elevated p62 level was found in chronic AF patients. These findings indicated impaired cardiac autophagy in chronic AF patients. Moreover, a reduction in LC3B expression in chronic AF patients, revealed by immunofluorescence staining, implied that the formation of autophagosomes was deceased. This finding further verified that there is impaired cardiac autophagy in chronic AF patients. Dual immunofluorescent staining confirmed the decreased engulfment of mitochondria into autophagosomes in atrial myocytes of patients with chronic AF.

Intact autophagic responses are crucial to preserving cardiovascular homeostasis in physiological conditions [8]. However, the role of autophagy in AF is still controversial. Yuan et al. [10] found activation of AMPK-dependent autophagy in AF patients and the rapid atrial pacing canine model. Recently, they reported that activation of autophagy contributed to AF by promoting degradation of L-type calcium channels [12]. Wiersma et al. [9] found that autophagy was activated upon endoplasmic reticulum (ER) stress and contributed to cardiomyocyte remodeling in experimental and human AF. Inhibition of ER stress was shown to protect against cardiac remodeling in in vitro and in vivo models of AF by preventing autophagy activation. However, a recent study including the largest clinical samples so far showed that impaired cardiac autophagy is associated with developing postoperative AF in patients after coronary artery bypass surgery [11]. In addition, another study revealed that ablation of NLRP3 inflammasome improved autophagy and reduced cardiac damage with protection of the prolongation of the age-dependent PR interval, which is associated with AF by cardiovascular aging [16]. How to interpret the contradictory results in these studies? We think that it is necessary to recognize the dynamic nature of autophagy. Shirakabe et al. [17] reported that autophagy was upregulated between 1 and 12 hours after transverse aortic constriction (TAC) in mice but was downregulated below physiological levels 5 days after TAC. This result suggests that autophagy is dynamic in the process of diseases. Activation of autophagy may be an adaptive response to pressure overload in the early stage, but autophagy inactivation after its temporary activation promotes the development of cardiac hypertrophy and heart failure [18]. The patients of the AF group enrolled in this study had long-standing persistent AF. The degree of atrial fibrosis is more severe in long-standing persistent AF [19]. Atrial fibrosis appears as a common endpoint in a variety of AF-promoting conditions, including senescence, heart failure, mitral valvular disease, and myocardial ischemia [20]. Recently, some evidence indicated that impaired cardiac autophagy was associated with cardiac fibrosis. Therefore, we speculate that autophagy is defective in the late stage of AF, and impaired autophagy may contribute to AF by prompting atrial fibrosis. The activated autophagy observed in AF may be an adaptive response to stress for maintaining cardiac homeostasis. However, it is harmful to cardiomyocytes when this response is excessive. The potential mechanisms on autophagy from adaptive to maladaptive response need to be investigated in the future. Therefore, we suggest that the role of autophagy depending on the stage of AF should be explored, and to which extent autophagy activation is beneficial for preventing AF should also be elucidated.

On the other hand, the autophagy process includes three parts: autophagy vesicle formation, engulfment of cargo, and fusion with lysosomes. At the beginning of autophagy, an autophagy vesicle with a double membrane is formed to encapsulate protein molecules or/and organelles that need to be degraded. The autophagic vesicles form autophagic bodies after wrapping the material to be degraded. Finally, lysosomes and autophagic bodies fuse into autophagic lysosomes, which use the internal hydrolases to degrade the encapsulated cell components [21]. Impaired function of the above three parts may cause autophagy instability and cell dysfunction. Mitochondrial homeostasis is an important mechanism for maintaining mitochondrial function in cells. Even in normal cells, mitochondria are also constantly fusing and dividing to maintain mitochondrial homeostasis.

Mitophagy is a special autophagy and plays a crucial role in the mitochondrial quality control and maintenance of mitochondrial function [8]. Early studies have confirmed that mitophagy can adjust the number of mitochondria in the cell to match the metabolic needs and, at the same time, remove the damaged mitochondria to complete the quality control of mitochondria [22, 23]. Hence, mitophagy is considered to be essential for maintaining cardiomyocyte homeostasis and viability in the basal state [24]. In the present study, increased mitochondria size and number were found in atrial myocytes of patients in chronic AF by transmission electron microscopy. These results indicated dysfunction in mitochondrial metabolism and quality control. Meanwhile, significantly increased autophagic vacuoles and decreased infusion autophagosomes with lysosomes were observed, which suggested the mitochondrial dysfunction may be associated with impaired mitophagy in atrial myocytes of patients in chronic AF. Increased expression of COX II and COX IV in the AF group further revealed that there was a defective mitophagy in atrial myocytes of patients with chronic AF. Consistent with our findings, Wiersma et al. [4] found myolysis and dispersed mitochondria in patients with persistent AF and reduced cellular ATP levels in patients with long-standing persistent AF. As a result, increased damaged mitochondria contributed to the generation of reactive oxygen species (ROS) via damage-associated molecular patterns (DAMPs) or the CAMK II pathway, which in turn regulated the cell function and led to enhanced activation of the NLRP3 inflammasome [25, 26]. Active NLRP3 inflammasomes could induce the release of inflammatory factors and production of AF-related extracellular matrix (such as collagen). In addition, ROS and oxidative stress are considered as a novel mechanism of AF [27]. Hence, elevated damaged mitochondria could be a key upstream target in the development of AF.

However, why did the mitophagy defect happen? The dual immunofluorescent stainings were used to investigate the underlying mechanism of mitophagy defect in chronic AF patients. Cox IV, LC3, and LAMP-1 were used for identification of mitochondria, autophagosomes, and lysosomes, respectively. The results showed that the fluorescence intensity caused by merge of Cox IV and LC3 was obviously decreased in the AF group, but the fluorescence intensity caused by merge of Cox IV and LAMP-1 was not significantly different between the SR group and the AF group. These findings suggested that mitophagy defects in atrial myocytes of patients with chronic AF may be due to dysfunction in the process of delivery of mitochondria into autophagosomes. However, our findings still need more in-depth studies to testify.

Collectively, our finding provided convincing experimental evidence, for the first time, that impaired mitophagy, during the phagocytosis of mitochondrial, is associated with human chronic AF. Mitophagy could be not only a potential therapeutic target for AF but also a promising mechanism of AF, which is suitable for further exploration.

## Figures and Tables

**Table 1:** The characteristics of patients with SR or AF.

Characteristics	SR group (*n* = 22)	AF group (*n* = 21)	*p* values
Age (years)	50.32 ± 10.14	53.29 ± 7.63	0.286
Gender (male)	12 (54.54)	9 (42.86)	0.443
Cardiac disease type			
MS	—	—	—
MI	3 (13.64%)	2(9.52%)	0.674
MS + MI	1 (4.55%)	—	—
AI	3 (13.64%)	—	—
CVL	15 (68.18)	19 (90.48)	0.155
Left atrial thrombus	1 (4.55%)	5 (23.81%)	0.095
LAD (mm)	44.91 ± 12.09	59.38 ± 12.19	*p* < 0.001
LVEF (%)	60.91 ± 12.16	59.90 ± 8.06	0.752
NYHA class			
I/II	2 (9.10)	2 (9.52)	0.961
III/IV	11 (50.00)	16 (76.19)	0.076

*Note*. Values expressed as mean ± SD or *n* (%). SR, sinus rhythm; AF, atrial fibrillation; MS, mitral stenosis; MI, mitral inadequacy; AI, aortic inadequacy; CVL, combined valvular lesion; LAD, left atrial diameter; LVEF, left ventricular ejection fraction; NYHA, New York Heart Association.

## Data Availability

The data used to support the findings of this study were supplied by Zhiyuan Jiang under license. Requests for access to these data should be made to Zhiyuan Jiang (e-mail: drjzy123@sina.com).
